# Assessing frequency and clinical outcomes of *BRCA* mutated ovarian cancer in Saudi women

**DOI:** 10.1186/s12885-021-09123-6

**Published:** 2022-01-03

**Authors:** Naela Agha, Bader Alshamsan, Sharifa Al-Farsi, Heba Aly Ateya, Fahad A. Almugbel, Hazem Abdullah Alotaibi, Ayman Omar, Amgad Shahin Mohamed, Hanan Alharthy, Tusneem Elhassan, Hany Salem, Hamed Alhusaini

**Affiliations:** 1 Medical Oncology, Oncology Center, King Faisal Specialist Hospital and Research Center, Riyadh, Saudi Arabia; 2 Northern Ireland Cancer Centre, Belfast City Hospital- Belfast- the UK, 66 Finaghy Road South, Belfast, BT10 0DE UK; 3 Department of Medicine, Qassim Medical College, Qassim University, Qassim, Saudi Arabia; 4 Surgical Oncology, Oncology Center, King Faisal Specialist Hospital and Research Center, Riyadh, Saudi Arabia; 5 National Cancer Institute, Cairo University, Cairo, Egypt; 6 Oncology center, Prince Mohmmad Medical City, Riyadh, Saudi Arabia; 7 Department of Clinical Oncology and Nuclear Medicine, Suez Canal University Hospitals, Ismailia, Egypt; 8 Department of Medicine, University of Maryland School of Medicine, Baltimore, MD USA

**Keywords:** Ovarian Cancer, BRCA1, BRCA2, Germline, BRCA mutation, Founder mutation

## Abstract

**Purpose:**

*BRCA* gene mutations (BRCAm) have an impact on patients’ characteristics and clinical outcomes of ovarian cancer (OC). The frequency and patterns of BRCAm vary among countries and ethnicities. There are limited data from Saudi Arabia (SA); thus, this study aims to determine the frequency, pattern, and impact on patient characteristics and outcomes of BRCAm OC compared to wild-type *BRCA* (BRCAw) in Saudi women.

**Methods:**

This retrospective study evaluated women diagnosed with non-mucinous OC, fallopian tube, or peritoneal carcinoma who had *BRCA* status tested in an accredited lab between January 2016 and December 2017. The associations between various parameters and BRCAm were estimated using logistic regression. Statistical analysis performed with SPSS (Version 27).

**Result:**

Sixty-one women with a median age of 52 at diagnosis were analyzed. Germline *BRCA* mutations were found in 41% of cases (25/61). The most common deleterious germline *BRCA1* mutation was c.1140dupG (39%). Most women (72%) had no family history of cancers and 82% had advanced stage. Regardless of *BRCA* mutations, an optimal overall response rate (ORR) to first-line treatment has been achieved although most cases relapsed (84%) and the majority were platinum-sensitive relapse (85%). Higher ORR to subsequent lines and better survival were obtained in women with *BRCA*-mutation.

**Conclusion:**

The prevalence of BRCAm of OC was higher in Saudi women compared to regional and most of the international figures. The better clinical outcomes of BRCAm women agreed with the reported evidence. Further studies on *BRCA* mutations of OC and genetic counseling are highly recommended.

**Trial registration:**

Trial approved by the Institutional Review Board of King Faisal Specialist Hospital and Research Center (RAC # 2171137) and conducted at King Faisal Specialist Hospital and Research Center, PO Box 3354, Riyadh 11,211, Saudi Arabia.

## Background

In high-income countries, ovarian cancer (OC) is the second most commonly diagnosed gynecological malignancy and the most common cause of death from it [1]. The age-standardized rate (ASR) incidence of OC worldwide is 3.9 per 100,000, and in Arab regions, it ranges between 0.9 and 8 per 100,000 [1, 2]. In Saudi Arabia (SA), OC ranks as the seventh most common cancer among females, with ASR of 3.7 cases per 100,000 and as the fifth leading cause of death, with an ASR of 2.5 deaths per 100,000. Worldwide OC ranked as the eighth most common cancer for incidence and mortality [3, 4]. In western countries, the inherited mutations in OC were found to be the cause for 14.1 to 18% of the OC cases and with the majority being caused by breast cancer associated gene mutations (BRCAm) [5, 6]. Furthermore, BRCAm increases the lifetime risk of developing OC from 1.3% in the general population to 44% (95% CI: 36–53%) in *BRCA1* and 17% (95% CI: 11–25%) in *BRCA2* mutation carriers [7]. A review of 173 women with breast cancer and OC from SA showed that the prevalence of germline BRCAm was 30.7% in OC and 10.2% in breast cancer [8]. In a recent systemic review from 22 Arab countries, six mutations were shown to be unique to the Saudi population: Four were located in *BRCA1* (c.1140dupG, c.5530delC, c.5054C > T, and c.711_712insTGAA), and two were located in *BRCA2* (c.2667delT and c.5760_5770del11) [2]. *BRCA1* and *BRCA2* are tumor suppressor genes that control cell growth and maintain genomic stability [9] . They are responsible for the repair of double-strand DNA breaks (DSBs) through the homologous recombination pathway, and a deficiency in *BRCA* function leads to genomic instability due to the inability to repair DNA damage through homologous recombination defect (HRD), thereby leading to tumorigenesis [10, 11]. Simultaneously, the mutation weakens tumor cells that can be targeted therapeutically [12] which explains the association between *BRCA* gene mutations and a better response to DNA-alkylating agents such as platinum in OC [10, 13]. Poly ADP-ribose polymerase enzyme (PARP) is essential for the repair of single-strand breaks of DNA. PARP inhibitors induce the synthetic lethality of cells with HRD, which occurs when there is a simultaneous mutation in two genes leading to cell death; however, no cell death occurs when the mutation is found in only one gene [12]. Many clinical trials have tested the efficacy of PARP inhibitors in the treatment of advanced OC. Early evidence showed improvement in objective response rate (ORR) and progression-free survival (PFS) in heavily treated patients, predominantly in those with either germline or somatic *BRCA* mutations as HRD occurs in germline *BRCA* mutations, somatic *BRCA* mutations, and *BRCA* promotor hypermethylation cases [14]. Moreover, this benefit has recently been proven to involve women with advanced high-grade serous OC after response to platinum-based chemotherapy (PBC) regardless of BRCA status [15]. The standard therapy for advanced epithelial OC is PBC following primary debulking surgery (PDS) [16]. Interval debulking surgery (IDS) is a feasible option after neoadjuvant chemotherapy and demonstrated similar survival outcomes as PDS [17]. However, more than 80% of patients experience disease recurrence after completing their treatment, with an unsatisfactory outcome to the second line of management [18].

The primary aim of this study was to measure the frequency and patterns of germline *BRCA* mutations among OC patients, to compare the clinicopathological characteristics, and to assess the clinical outcomes of *BRCA* mutant vs *BRCA* wild-type patients.

### Methods

A retrospective study was conducted on all patients diagnosed with primary ovarian, fallopian tube, or peritoneal carcinoma who were tested for *BRCA* mutations and followed up at the King Abdullah Oncology Centre at the King Faisal Specialist Hospital and Research Center (KFSH&RC) between January 2016 and December 2017. Women with pathological confirmation of serous carcinoma, clear cell carcinoma, and endometrioid carcinoma were eligible, whereas those with borderline cancer and mucinous carcinoma were excluded. Germline *BRCA* mutations were obtained from Myriad Genetic Laboratories Inc., which was certified by the College of American Pathologists and Clinical Laboratory Improvement Amendments as per the National Comprehensive Cancer Network (NCCN) guidelines [19]. This project was conducted in accordance with the ethical principles in Helsinki’s Declaration (2000) and was approved by the Institutional Review Board of KFSH&RC (RAC # 2171137). The clinical information collected from the medical records included age, personal and family history of cancer, tumor histology, grade, Federation of Gynecology and Obstetrics (FIGO) stage, CA125 level, and *BRCA* status. In addition to lines of chemotherapy, best response, time to progression, platinum sensitivity, and status at last follow-up were evaluated. The tumors were staged according to the 2017 8th Edition of the American Joint Committee on Cancer and the FIGO classification system. The patients were followed up based on KFSH&RC guidelines (every two to three months for the first two years and then every six months). At each visit, clinical assessment and serum CA125 test were performed. The abdominal ultrasound scans were performed every six months, and computed tomography of the chest and abdomen was done every year for five years unless relapse was suspected. Platinum-sensitive relapse was defined as tumor relapse that occurred more than six months after completion of the last cycle of PBC. Tumor response was assessed based on the Response Evaluation Criteria in Solid Tumors Version 1.1 (RECIST 1.1). The clinical outcomes investigated were overall response rate (ORR) to first and subsequent lines, disease-free survival (DFS), and overall survival (OS). The ORR has been defined as the sum of partial and complete responses divided by the total number of patients. DFS was defined as the interval between histologic diagnosis and first progression, death as a result of disease, or last follow-up. OS was defined as the interval between histologic diagnosis and the date of death as a result of disease or last follow-up.

### Statistical analysis

Categorical values were described as frequencies compared with Chi-square test or Fisher’s exact test, and continuous values were described as the median with interquartile range (IQR) and compared using the Mann-Whitney U test. Associations between various parameters and the *BRCA* mutations were estimated by logistic regression. The Kaplan–Meier estimator was used to determine DFS, and OS and survival curves were compared using the log-rank test, and a multivariant analysis was conducted using the Cox regression. All variables were tested for the affirmation of the proportionality assumption. Variables that violated the proportionality assumption were entered as time-dependent covariates. *BRCA* mutations were considered the main effect and were kept in the model at all times. Interactions between *BRCA* mutations and other significant variables were evaluated, and significant interactions were considered. A *p*-value of ≤0.05 was considered statistically significant. The SPSS for Mac, v27; IBM Corp, Armonk, NY, USA, was performed for statistical analysis.

## Results

A total of 61 women were eligible for analysis. The median age at diagnosis was 52 years (IQR: 44–61.5). *BRCA* mutations were found in 25 women (41%), including 23 with *BRCA1* mutations and 2 with *BRCA2* mutations. The patient and disease characteristics stratified by *BRCA* status are shown in Table 1. The main presenting symptom was abdominal distension (35 patients, 57.3%). The logistic regression revealed a significant association between family history of malignancy and *BRCA* mutations (*p* = 0.03). However, *BRCA* mutations were not statistically significantly associated with age and stage at diagnosis, patient region, or history of primary cancer.

**Table 1:** Patient, disease, and treatment characteristics stratified by *BRCA* status (*n* = 61)

Characteristics		*BRCA* mutant *n = 25 (41%)*	*BRCA* wild type *n = 36 (59%)*	*P*-Value*
		N (Frequency)	N (Frequency)	
Age at diagnosis				
Median (IQR)		50 (43–56)	55 (46–66)	0.13
Age ≤ 50		14 (56)	15 (41.7)	0.27
Positive personal history of cancer		5 (20)	1 (2.9)	0.04
Positive family history of cancer		7 (28)	2 (5.6)	0.02
Presence of comorbidities*		19 (76)	24 (66.7)	0.43
Histology	High-grade serous	25 (100)	35 (97.1)	0.39
	Endometrioid		1 (2.9)	
High grade FIGO stage		25 (100)	34 (94.4)	0.48
	Stage 1	6	3	0.65
	IA	2	0	
	IB	2	1	
	IC	2	2	
	Stage 2	1	4	
	IIA	1	1	
	IIB	0	3	
	Stage 3	15 (58)	21 (55)	
	IIIA	0	2	
	IIIB	1	2	
	IIIC	14	17	
	Stage 4	4	10	
High CA125 (> 35)		20 (80)	26 (72.2)	0.46
Initial management	PDS	12 (48)	18 (50)	0.87
	NAC	13 (52)	18 (50)	
No. of lines, median (IQR)		3 (1–5)	3 (2–4.75)	0.79
Lines of treatment				
First line (n = 61)		(n = 25)	(*n* = 18)	
PBC Non PBC		100%	100%	0.26
Second line (*n* = 49)		(n = 18)	(*n* = 31)	
PBC		88.9	83.4	0.79
Non PBC		11.1	16.6	
Third line (*n* = 39)		(*n* = 13)	(*n* = 26)	
PBC		73.1	64	0.42
Non PBC		26.9	36	
Fourth line (*n* = 23)		(*n* = 10)	(n = 13)	
PBC		50%	23.1%	0.38
Non PBC		50%	76.9%	
Fifth line (n = 13)		(*n* = 7)	(*n* = 5)	
PBC		–	–	0.68
Non PBC		100%	100%	

Categorical values were compared with the Chi-square test or Fisher’s exact test, and continuous values were described as the median with interquartile range (IQR) and compared using the Mann-Whitney U test

Comorbidities: hypertension, DM, hypothyroidism, bronchial asthma, dyslipidemia, or osteoarthritis. PBC; platinum-based chemotherapy; non PBC, non-platinum-based chemotherapy (Paclitaxel, Liposomal doxorubicin, Etoposide, Gemcitabine, Topotecan, Letrozole, Tamoxifen)

There were 15 different pathogenic variants identified, including 13 with *BRCA1* and 2 with *BRCA2*. The three most common deleterious germline *BRCA1* pathogenic variants were c.1140dupG (9 patients, 39%), c.5530del (3 patients, 13%), and c.5095C > T (2 patients, 8%). The other pathogenic variants were each observed once. Table 2 presents all pathogenic variants of the mutated genes, age, geographical region of the patients, and personal and family histories of cancer. The univariant analysis revealed no association between c.1140dupG, the most common deleterious mutation, and age, stage at diagnosis, relapse rate, platinum sensitivity, or patient region; however, all the OC cases from the western province (3 patients) and 29% (4 patients) from the central region carried the c.1140dupG pathogenic variant of the *BRCA1* gene mutation. Of the *BRCA* wild-type patients, two had a positive family history of cancer, including a mother with a brain tumor and a sister with colon cancer; one patient had a personal history of cervical cancer.

**Table 2:** Patients, age and regions, family history, and deleterious mutations (*n* = 25)

Age	Region*	Personal History of cancer	Family members/ type of cancer	Gene	Mutation	Protein change
60	South	–	–	*BRCA1*	c1140dupG	p.Lys381Glufs*3
45	Central	Breast Ca	–	*BRCA1*	c.1140dupG	p.Lys381Glufs*3
52	Western	Breast Ca	Sister (breast)	*BRCA1*	c.1140dupG	p.Lys381Glufs*3
52	Central	–	–	*BRCA1*	c.1140dupG	p.Lys381Glufs*3
48	Western	–	–	*BRCA1*	c.1140dupG	p.lys381Glufs*3
40	Eastern	–	–	*BRCA1*	c1140dupG	p.Lys381Glufs*3
45	Central	–	Sister (breast/ovarian) Father (Lung)	*BRCA1*	c.1140dupG	p.Lys381Glufs*3
43	Central	Breast Ca	Mother and Sister (breast)	*BRCA1*	c.1140dupG	p.Lys381Glufs*3
41	Eastern	–	–	*BRCA1*	c.1140dupG	p.Lys381Glufs*3
59	Northern	–	–	*BRCA1*	c.5530del	p.Leu1844Serfs*11
67	Central	–	–	*BRCA1*	c.5530del	p.Leu1844Sarfs*11
49	Central	Breast Ca	–	*BRCA1*	c.5530del	p.Leu1844Serfs*11
56	Central	–	Sister (breast)	*BRCA1*	c.5095C > T	p.Arg1699Trp
69	Southern	–	–	*BRCA1*	c.2572C > T	p.Gln858*
48	Central	Pheochromocytoma, Breast Ca	Brother (colon Ca)	*BRCA1*	c.2405_2406del	p.Val802Glufs*7
38	Central	–	–	*BRCA1*	c.2410_2413del	p.gln804Valfs*10
56	Southern	–	Sister (breast)	*BRCA1*	c.1426_1433del	p.His476*
62	Northern	–	–	*BRCA1*	c.5074 + 2 T > T	
50	Northern	–	–	*BRCA1*	c.5095C > T	p.Arg1699Trp
53	Central	–	Sister (breast/ovarian) Father (Colon)	*BRCA1*	c.135-1del	
43	Central	–	–	*BRCA1*	c.1016del	p.Lys339Argfs*2
56	Central	–	–	*BRCA1*	c.69del	p.Glu23Valfs*17
35	Southern	–	–	*BRCA1*	c.708_711dupTGAA	p.His228*
50	Northern	–	–	*BRCA2*	c.7007G > A	p.Arg2336His
41	Central	–	–	*BRCA2*	c.5762_5772del	p.Phe1921Serfs*3

Regions according to the Saudi cancer registry: Central region (Riyadh, Qassim, and Hail), Northern region (Madinah, Tabuk, Jouf, and Northern), Western region (Makkah, Madinah, Jeddah, and Taif), Eastern region (Dammam and Ahsa), and Southern region (Jizan, Naran, Baha, and Asir

All women in this cohort underwent debulking surgery and received chemotherapy during their treatment; 49% received PDS and then adjuvant chemotherapy, and 51% started with neoadjuvant chemotherapy and IDS with no statistical difference (*p* = 0.98). All women received PBC as the first-line treatment. A total of 87% of both groups received a regimen consisting of IV cisplatin and paclitaxel every 3 weeks. The patients received a median of three chemotherapy lines; specifically, 21, 17, and 62% of patients received one, two, and three or more chemotherapy lines, respectively. The platinum sensitivity dropped with subsequent lines; 14, 41, and 73% of women received non-platinum-based therapy as the second-line, third-line, and fourth-line treatments, respectively, and 100% (13 patients) received non-platinum-based therapy as the fifth-line treatment. The ORR to the first line of management was 100%, with a higher complete response (CR) in *BRCA* mutant women than in wild-type women (92% vs 72.7%, *p* = 0.08). The relapse rate was 84%, and the majority (85%) were platinum-sensitive. Additionally, 84% of *BRCA* mutant vs. 80% of *BRCA* wild-type patients experienced platinum-sensitive relapse after first-line therapy (*p* = 0.43). In the subsequent lines of treatment, the ORR also was higher in the *BRCA* mutant group compared with the *BRCA* wild-type group: second-line (94.4% vs 64.5%, *p* = 0.01), third-line (84.7% vs 30.6%, *p* = 0.002). The ORR did not reach statistical significant in fourth-line therapy (40% vs 22.2%, *p* = 0.40) (Table 3). Thirteen out of 25 women with *BRCA* mutations received a PARP-inhibitor, namely Olaparib, and over half of the patients received Olaparib after third relapse. (54%, *n* = 7). Olaparib was discontinued due to disease progression in eleven patients and anemia in one patient. Olaparib treatment was still ongoing for one patient. The sample size limited further analysis.

**Table 3:** Response rate to different lines of chemotherapy based on BRCA status

Best Response	First line (*n* = 61)		Second line (*n* = 49)		Third line (*n* = 39)		Fourth line (*n* = 23)	
	BRCAm *n* = 25	BRCAw *n* = 36	BRCAm *n* = 18	BRCAw *n* = 31	BRCAm *n* = 13	BRCAw *n* = 26	BRCAm *n* = 10	BRCAw n = 13
CR	92%	72.7%	50%	29%	46.2%	7.7%	20%	7.7%
PR	8%	27.3%	44.4%	35.5%	38.5%	19.2%	20%	7.7%
SD	–	–	5.6%	9.7%	–	26.9%	10%	15.4%
PD	–	–	–	25.8	15.4%	34.6%	50%	38.5%
NA	–	3 patients	–	–	–	3 patients	–	4 patients
ORR	100%	100%	94.4%	64.5%	84.7%	30.6%	40%	22.2%
P-Value*			**0.01**		**0.002**		**0.40**	

* Chi-square or Fisher’s exact test; *CR*, complete response; *PR*, partial response; *SD*, stable disease; *PD*, progressive disease; *NA*, not available; *ORR*, objective response rate

**Fig. 1:**
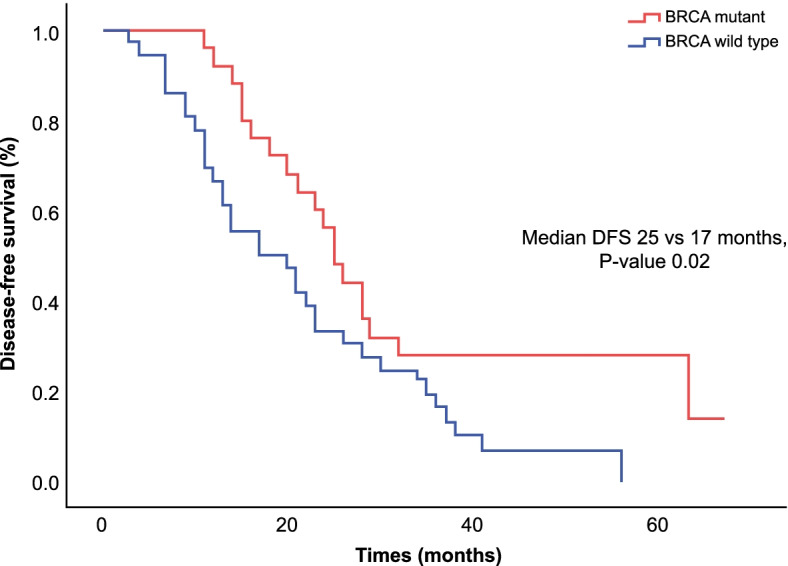
Disease-free survival of ovarian cancer stratified by *BRCA* status for first-line therapy. The Kaplan–Meier estimator was used to determine DFS among patients with germline *BRCA* mutation and those without germline *BRCA* mutation. Two sided *P* values were calculated with the use of the stratified log rank test and CI denotes confidence interval. The median DFS was longer in the *BRCA* mutant women 25 (95% CI: 21.7–28.2) vs. 17 (95% CI: 8.7–25) months, (*p* = 0.02)

**Fig. 2:**
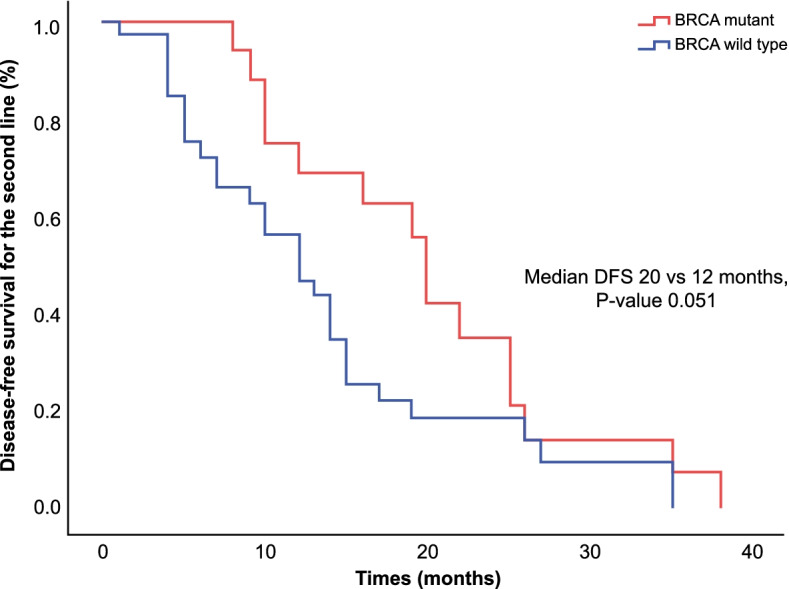
Disease-free survival of ovarian cancer stratified by *BRCA* status for second-line therapy. The Kaplan–Meier estimator was used to determine DFS for second line therapy among patients with germline *BRCA* mutation and those without germline *BRCA*. Two sided P values were calculated with the use of the stratified log rank test and CI denotes confidence interval. The median DFS of second-line treatment (50 patients) in the *BRCA* mutant group was 20 months (95% CI: 18.2–21.7) vs. 12 months (95% CI: 7.8–16.1) in the wild-type group (*p* = 0.051)

**Fig. 3:**
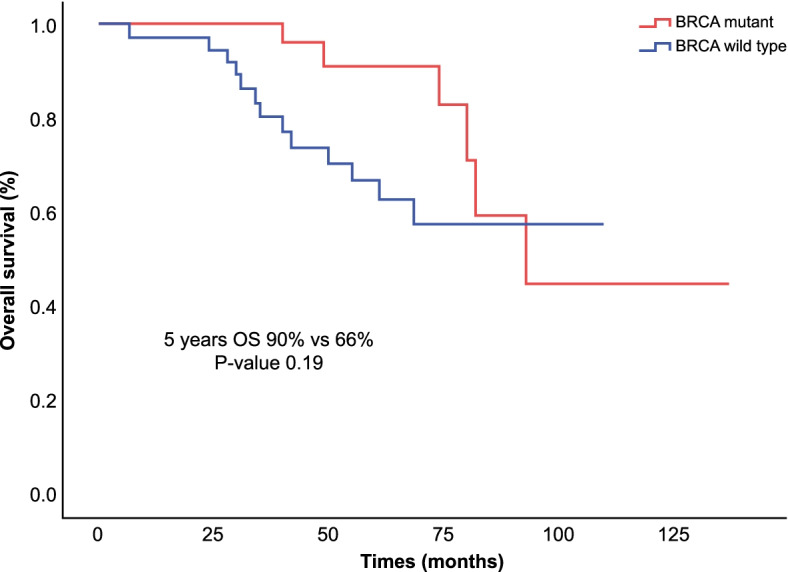
Five-year overall survival of ovarian cancer stratified by *BRCA* status. The Kaplan–Meier estimator was used to determine OS among patients with germline *BRCA* mutation and those without germline *BRCA* mutation. Two sided P values were calculated with the use of the stratified log rank test and CI denotes confidence interval. The median OS was not reached. However, the five-year OS rate for *BRCA* mutant patients was 90.9% vs. 66.7% for wild-type patients (*p* = 0.19)

The median follow-up duration was 59 months (range: 7–93). The date of diagnosis of the last patient enrolled in this cohort was on December 2017 and the date of last follow up was on August 2020. The median DFS was longer in the *BRCA* mutant women 25 (95% CI: 21.7–28.2) vs. 17 (95% CI: 8.7–25) months, *p* = 0.02) (Fig. 1). The Cox regression analysis for DFS adjusted by age and comorbidities was statistically significant for *BRCA* mutant vs. wild-type patients (hazard ratio (HR) = 0.52, 95% CI: 0.23–0.92, *p* = 0.02). The median DFS of second-line treatment (50 patients) in the *BRCA* mutant group was 20 months (95% CI: 18.2–21.7) vs. 12 months (95% CI: 7.8–16.1) in the wild-type group (*p* = 0.051) (Fig. 2). The median OS was not reached. However, the five-year OS rate for *BRCA* mutant patients was 90.9% vs. 66.7% for wild-type patients (*p* = 0.19) (Fig. 3).

## Discussion

This is the first study to compare the clinical characteristics and clinical outcomes of BRCAm and BRCAw in Saudi women with OC. The research highlights the higher prevalence, the better clinical outcomes of *BRCA* mutants, and the importance of early access for *BRCA* testing and treatment. “The frequency of *BRCA*-associated OC was higher in this current cohort than in a previous study (40% vs. 30%). This could be explained by the higher association of *BRCA* with high-grade serous carcinoma [5], almost exclusively all patients were high-grade serous carcinoma vs. 79% was serous carcinoma in the previous report [8]. The factors related to the high prevalence of *BRCA* pathogenic variants in Saudi women remain unknown. The frequency in this cohort is close to the *BRCA* gene mutations rate found among Italian women (39.2%) diagnosed at a median age of 50 years with high-grade serous carcinoma [20]. Also, it is close to the reported rate in Ashkenazi Jewish (41%) and another cohort of Italian women (43.5%); however, the serous carcinoma were in 68 and 56% of patients, respectively [21, 22]. The most common mutations in this study are c.1140dupG, c.5530del, and c.5095C > T, which agrees with recent reports [2, 8]. However, the most common mutations in the Ashkenazi are c.68_69del and 5266dup [21], and those in the Italian women are c.3756_3759del and c.1360_1361del [20, 22]. The higher percentage of *BRCA1* mutations compared with *BRCA2* mutations (92% vs 8%) in this study agrees with previous publications although those studies reported higher percentages of *BRCA2* mutations [5, 22]. This trend is reversed in some Asian populations, with a higher percentage of *BRCA2* mutations compared with *BRCA1* mutations [23]. Clearly, the pattern and frequency of *BRCA* mutations vary significantly in relation to race/ethnicity and geographical location [24].

The median age at diagnosis was lower than that in other regions: 52 vs 63 years; however, there was no statistically significant difference between BRCAm and BRCAw (*p*-value = 0.13) [25]. There was a greater association of a family history of malignancy and a personal history of cancer with the *BRCA* mutant patients than in the wild-type patients (*p* = 0.02 and *p* = 0.04, respectively), which has been reported earlier [26]. However, most cases of OC did not have a positive family history, as noted in earlier studies [5]. Therefore, the NCCN guidelines recommend susceptibility gene screening regardless of family history for all epithelial OC cases, including fallopian tube cancer or peritoneal cancer diagnosed at any age [19], which has been followed in many institutions [22]. Agreeing with previous studies, abdominal distension the most common presenting symptom in this cohort, and there was no significant difference between BRCAm and BRCAw in terms of FIGO stage at presentation and CA-125 level [5, 27].

The higher ORR to subsequent lines of treatment in the *BRCA* mutant group agrees with earlier evidence showing that *BRCA* mutations increase the susceptibility of the cells to be destroyed by chemotherapy [13]. All patients received intravenous doublet PBC in a first-line setting, in agreement with the international standard of care [28, 29] Intraperitoneal chemotherapy is not approved at KFSHRC as the standard treatment because of its high toxicity profile and worsening quality of life [29, 30].

The higher DFS and OS in *BRCA* gene mutations have been shown in several previous studies, and this finding corresponds to a better response to chemotherapy owing to the deficiency of mechanisms of DNA repair [31]. This trend was clear in this cohort, which had a prolonged median DFS in the *BRCA* mutant group (25 vs. 17 months) in the first-line setting, which was statistically significant. Additionally, the median DFS after second-line treatment was eight months longer in the *BRCA* mutant group (Fig. 2), and the five-year OS was 90% vs 66% (Fig. 3) in the *BRCA* mutant group compared to *BRCA* wild group; however, this difference was not statistically significant, However, longer follow-up is necessary.

We acknowledge that the small sample size likely yielded underpowered comparisons. Moreover, these patients were recruited from a tertiary referral center and therefore may not be representative of patients at primary cancer centers, although they were obtained from the largest referral hospital in the region. Furthermore, the high *BRCA* pathogenic variants prevalent in the current study among relatively young age and predominantly high-grade serous carcinoma should be interpreted carefully as that may not represent a population-based sample.

## Conclusions

*BRCA* gene mutations in Saudi women with OC predominantly involve the *BRCA1* gene. The founder mutation was c.1140dupG, which was observed in more than one-third of the cases. BRCAm women had a better ORR in subsequent lines of therapy and a longer DFS than the BRCAw-type women.

## Data Availability

The datasets used and analyzed during the current study are available from the corresponding author on reasonable request.

## Abbreviations

ASR: Age-standardized rate
CR: Complete response
DFS: Disease-free survival
DSB: Double-strand breaks
EOC: Epithelial ovarian cancer
HRD: Homologous recombination defect
IDS: Interval debulking surgery
KFSH: King Faisal Specialist Hospital
NCCN: National Comprehensive Cancer Network
OC: Ovarian cancer
ORR: Objective response rate
OS: Overall survival
PARP: Poly ADP-ribose polymerase
PFS: Progression-free survival
SA: Saudi Arabia
FIGO: International Federation of Gynecology and Obstetrics
PBC: Platinum-based chemotherapy
PDS: Primary debulking surgery
